# Stubby or Slender? Ear Architecture Is Related to Drought Resistance in Maize

**DOI:** 10.3389/fpls.2022.901186

**Published:** 2022-06-13

**Authors:** Xian-Min Chen, Feng-Yuan Li, Shuai Dong, Xin-Fang Liu, Bin-Bin Li, Zu-Dong Xiao, Tao Deng, Yan-Bo Wang, Si Shen, Shun-Li Zhou

**Affiliations:** ^1^College of Agronomy and Biotechnology, China Agricultural University, Beijing, China; ^2^Corn Research Institute, Liaoning Academy of Agricultural Sciences, Shenyang, China; ^3^Innovation Center of Agricultural Technology for Lowland Plain of Hebei, Wuqiao, China

**Keywords:** maize, drought resistance, ear architecture, silking, yield

## Abstract

Ear architecture is determined by two stable heritable traits, kernel row number (KRN) and kernel number per row (KNPR), but its relationship with drought resistance is still vague. To this end, we obtained 16 and 11 hybrids with slender (less KRN but more KNPR) and stubby (more KRN but less KNPR) ears by intentionally crossbreeding, respectively. These hybrids were exposed to a seven-day water deficit (WD) since silk emergence coupled with synchronous (SP) and continuous pollination (CP) to alter the pollination time gaps on ears. The results showed that the emerged silks in CP were 9.1 and 9.0% less than in the SP treatment in the stubby and slender ears, respectively, suggesting the suppression of asynchronous pollination on silk emergence. The stubby ears performed higher silking rate and yield compared with the slender ears with or without drought stress. To eliminate the inherent difference in sink capacities, we selected four hybrids for each ear type with similar silk and kernel numbers for further analyses. Interestingly, the stubby ears were less affected in silking rate and thus performed higher yield under drought compared with the slender ears. The finding suggests that ear architecture matters in the determination of drought resistance that deserves more attention in breeding.

## Highlight

Maize hybrids with the stubby ears, obtained by the designed cross of inbred lines, performed faster silking rate and stronger drought resistance compared with those hybrids with the slender ears.

## Introduction

Maize (*Zea mays* L.) was domesticated from teosinte (*Zea luxurians*) with only two rows nearly 9,000 years ago (Matsuoka et al., 2002). In domestication, increasing kernel row number (KRN) and kernel number per row (KNPR) promote the kernel number per ear and greatly improve the yield of the modern maize (Doebley, 2004; Peng et al., 2011; Wang et al., 2011). As the two traits have higher heritability and better stability, they became the primary breeding traits for yield improvement to feed the growing population (Messmer et al., 2009; Li et al., 2013; Du et al., 2021; Ning et al., 2021). Since the 1960s, ear rows of single-cross varieties have increased and stabilized around 16 rows in China (Duvick et al., 2004; Qin et al., 2016). Pairwise, an agricultural biotechnology company, has launched field trail in the US Midwest, with backing from Bayer, of CPISPR-edited maize with more than 16 rows on an ear, to increase efficiency and boost yield in maize production (Sheridan, 2021). However, ear length in China is significantly reduced over time in recent years (Qin et al., 2016; Chen et al., 2021). Domestication, breeding, and genetic modification, representing different routes to obtain genetic changes of desired traits of germplasm, have all targeted on KRN and length of maize ear, emphasizing the importance of these traits in yield improvement. Presciently, continuous selection of germplasms with more rows and less ear length would inevitably turn the ear architecture from slender to stubby. Nevertheless, how the resultant changes in ear architecture affect the resistance to environmental stress is still less understood, which deserves more attention under the scenarios of global climate change.

Drought has become one of the most severe threats to agriculture under global warming (Lawlor, 2013; Wheeler and Braun, 2013). Most crops are especially sensitive to water deficit during flowering stage, when pollination and fertilization occur and fruit or seed establishes for yield potential (Barnabas et al., 2008; Fang et al., 2010; Zhang et al., 2014; Parent et al., 2017). Through different underlying mechanisms, drought stress at the flowering stage may affect reproductive success and the eventual yield output (Shen et al., 2020, 2022). Commonly, drought-induced leaf senescence, stomatal closure, and photosynthesis suppression would reduce assimilate supply for sink establishment (Boyle et al., 1991; Li et al., 2018; Sade et al., 2018). Subsequent changes in metabolic pathways and the increased competition for assimilates lead to low carbon efficiency for the ovary (Ruan et al., 2012; Oury et al., 2016a; Shen et al., 2020). Interestingly, maize, a monoecious plant, possessing unisexual male (tassel) and female flowers (silks) in physically separated parts of the plant, is more susceptible to water condition during flowering period than all other crops with hermaphrodite flowers, including wheat and rice (Borrás et al., 2007). Due to the spatial separation, and respective development, of tassel and silks, drought stress may cause maize yield loss through other mechanisms in addition to suppression of the source (Shen et al., 2020). Typically, water deficit severely inhibits genes associated with expansive growth and cell division, suppresses silk growth, and extends the anthesis-silking interval (ASI), leading to pollination failure of silks and consequent losses in seed number (Edmeades et al., 1993; Fuad-Hassan et al., 2008; Verbraeken et al., 2021). Hence, shorter ASI has been a critical trait for the breeders to select drought-resistant varieties (Edmeades et al., 1993; Bolaños and Edmeades, 1996; Bruce et al., 2002).

Other than the discordance in tasseling and silking of maize plant, intra-organ growth cooperation in response to drought stress determines yield performance in many species (Rocha and Stephenson, 1991; Hays et al., 2007; Chen et al., 2013; Shen et al., 2018). Interestingly, silks initiated from different ear regions grow asynchronously, and the inherent orders of differentiation and elongation of silks are from the basal to the apical region of an ear. The silks from the basal ear, however, are the furthest from the top end of the bracts. As a result, firstly emerged silks are usually from the ovaries at positions of five to eight rings on an ear and then sequentially from both sides, and the silks from the apical region of an ear are the latest to emerge (Oury et al., 2016b). In general, the time intervals between the earliest and latest emerged silks would reach about 4 to 8 days under well-water (Westgate and Westgate, 1993). The asynchronous emergence of silks results into asynchronies in pollination and fertilization for grain development within ears, thus manipulating the competition for assimilate among siblings (Oury et al., 2016b; Shen et al., 2018). Recently, a study demonstrated that pollination time gap (PTG) between the first and last emerged silks was one of the main factors inducing kernel abortion on the apical ear (Shen et al., 2018). Moreover, the frequency of kernel abortion at different ear positions was negatively correlated with the base-to-apex gradient pattern of silk emergence, which depends on the position of the ovaries and does not change even under drought stress (Oury et al., 2016b). These studies emphasized the importance of synchronous development for kernel set on different ear positions. Notablely, the distance for silks emerge from the bracts is shorter on the stubby than the slender ears. However, the silking process and PTGs of different ear types and their performances under drought stress are still unclear.

To understand how ear architecture influences silk emergence, PTGs, and yield in response to drought stress, this study obtained 16 and 11 hybrids with the slender (less KRN and more KNPR) and the stubby (more KRN and less KNPR) ears, respectively, by purposeful crossing of inbred lines. On these hybrids, two manual pollination treatments, continuous pollination and synchronous pollination, were applied to simulate asynchronous pollination of the emerging silks and to eliminate the PTG, respectively. By measuring the dynamic in silk emergence at flowering stage and yield performance at maturity, this study tested the hypothesis that ear architecture may be related to silking dynamics, PTGs, and thus yield in response to drought stress. By these data, the relationship between ear architecture and drought resistance were analyzed. These findings provide new insight into the trait of ear architecture for further breeding of drought-resistant varieties.

## Materials and Methods

### Plant Materials

To obtain hybrid materials with the slender and stubby ears, we applied artificial hybridization within inbred lines with > 8 rows and those with < 24 rows, respectively. In these F1 hybrids, we further obtain 27 hybrids with slender (16 hybrids) and stubby ears (11 hybrids) based on KRN and KNPR for the subsequent experiments. Cultivation and manual crossing of inbred lines were conducted at the South Experimental Site of Liaoning Academy of Agricultural Sciences (Liaoning Province, China, 123.3°E, 41.5°N). Specifically, inbred lines of KS2, KS4, XL21, and H2671 were the female parent, and 95C189-2, 92C0003-10, 95C914-3, 95C369, 95C545-2, and 95C453-2 were the male parent, crossed for F1 hybrids with < 16 ear rows. Inbred lines of D598B, D598, D360, L598, 94C1476-2, and 94C1546-1 were the female parent, and 94C1626-1, 94C1368-1, and 94C1349-3 were the male parent, crossed for F1 hybrids with > 16 ear rows. Each inbred lines of female parent were sown for eight rows, and male parent were sown for one row on April 2019. Rows for each inbred lines were 5-m long and 0.65-m wide. The field was managed under conventional cultivation. F1 seeds were collected in September 2019.

### Plant Growth and Management

A total of 27 hybrids with the stubby or slender ears were sown on 26 May, 2020, at the Wuqiao Experimental Station of China Agricultural University (Hebei Province, China, 116.3°E, 37.4°N). Seeds with the same weight were selected and sown in dibble seedling raising (48 mm side length of edge × 18 mm side length of bottom × 93 mm height) and supplied with sufficient water to ensure germination. At the V2 stage, uniformed maize seedlings were transplanted into plastic pots (30 cm height × 17 cm radius) with 15 kg of dry sand soil (silt loam), adequate moisture, and 11 g of basal compound fertilizers (N 15%, P_2_O_5_ 15%, K_2_O 15%). Topdressing 5 g urea (N 46%) was applied at the V12 stage. Each treatment consisted of five plants from independent pots as biological replicates. Maize plants were grown outdoors under the movable shelter to prevent rainfall. Artificial irrigation was applied to maintain soil water content. Pesticide was applied to prevent insects throughout the growth period.

### Manual Pollination and Water Treatments in Silking Period

**Figure 1:**
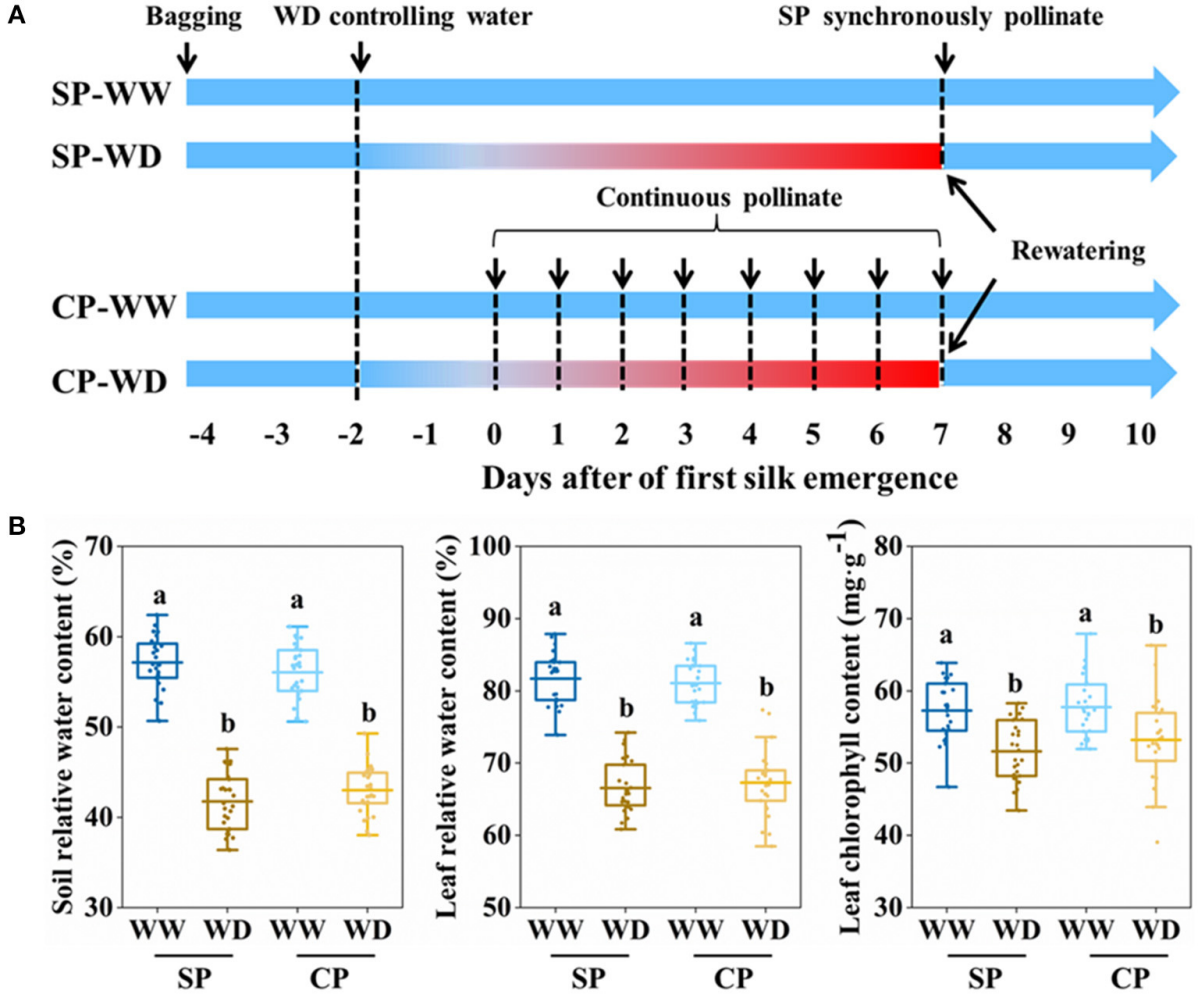
Processes of manual pollination and control of soil water content. **(A)** Summary of the pollinatioin time and water control. **(B)** Soil relative water content, leaf relative water content, and leaf chlorophyll content in all treatments at 3 days after of first silk emergence. SP, synchronous pollination; CP, continuous pollination; WW, well-watered treatment; WD, water-deficit treatment. One-way ANOVA followed by Duncan's new multiple range test, *n* ≥ 3; letters (a and b) indicate significant differences (*p* < 0.05).

All emerged ears were bagged before silking to prevent natural pollination. Synchronous pollination (SP) and continuous pollination (CP) were applied to eliminate pollination time gaps (PTGs) and simulate natural pollination, respectively (Figure 1A). In SP treatment, all silks were hand-pollinated with fresh pollen after silks fully emerged (~7 days after first silk emergence); in CP treatment, silk cluster was daily pollinated with fresh pollen at 10:30 am, from the first day when silk emerged until 7 days after silk emergence. Fresh pollens were collected from well-watered plants at 10:00 am.

Soil moisture was controlled by artificial irrigation and movable shelter that prevented rainfall. Before treatment, all plants were daily watered to maintain the soil relative water content (SRWC) above 75% (Figure 1A). In water-deficit (WD) treatment, irrigation was ceased at about 2 days before silking to allow the SRWC rapidly decrease to reach water deficit at silk emergence, then limited irrigation (0.25 L·Pot^−1^·day^−1^) was applied from 1 to 7 days after silk emergence, while plants in well-watered (WW) were sufficient irrigated (1 L·Pot^−1^·day^−1^). At 7 days after silk emergence, sufficient irrigation was recovered for WD and maintained during the rest filling stages (Figure 1A).

### Measurements of Soil Relative Water Content, Ear-Leaf Relative Water Content, and Chlorophyll Content

The soil bulk density (SBD) and saturation moisture capacity (SSMC) of the three samples of upper soil (5–10-cm soil layer) were measured by the cutting-ring method before silking. The soil volumetric water content (VWC) in the upper layer of each potted (5–10-cm soil layer) was measured three times by the soil moisture sensor (ThetaProbe ML2x, Delta-T Device). The soil relative water content (SRWC) was calculated by the following formula:


$$\begin{array}{l} SRWC\;\left(\%\right)=\frac{VWC\;{{\left(\%\right)}^{*}\rho }_{H_{2}O}\;\left(g.cm^{-3}\right)}{SBD{\left(g.cm^{-3}\right)}^{*}SSMC\left(\%\right)}*100\% \end{array}$$

The middle part of the ear leaf was collected on the third day after silking. Each sample was divided into two parts: one part (3 × 3 cm) was immediately weighted for fresh weight (Wf) and then put into distilled water to absorb water for 16 h for the measurement of saturated leaf weight (Wt). Afterward, leaf sample was dried at 80 °C for 48 h until constant weight in an oven and weighed with dry weight. The leaf relative water content (LRWC) was calculated by the following formula:


$$\begin{array}{l} LRWC\;\left(\%\right)=\frac{Wf-Wd}{Wt-Wd}*100\% \end{array}$$

The another part of fresh leaf (2 × 2 cm) was cut into pieces and soaked in 8 mL of 95% alcohol for 36 h in darkness to extract chlorophyll, and the OD values were measured at the wavelengths of 665 nm and 649 nm with a spectrophotometer (UV9100, LabTech). The concentrations of chlorophyll a (Ca) and b (Cb), and chlorophyll content were calculated according to the method of Arnon (1949):


$$\begin{array}{c} Ca=13.95^{*}D665-6.88^{*}D649\\ Cb=24.96^{*}D649-7.32^{*}D665\\ Leaf\;chlorophyll\;content\;\left(mg\cdot cm^{-3}\right)=\;\frac{{\left(Ca+Cb\right)}^{*}V}{Area} \end{array}$$

### Silking Dynamics

Silking dynamics is that the number of silks counted every day after the first silk emerged out of the husk, until silk emergence ceased. Silks were manually counted with tweezers and taken care to avoid breaking the ovary–silk junctions.

The duration of silk emergence was from first silk emergence to 90% of the silk emergence. Silking dynamics was evaluated by the parameters of silking rate, and the silk number of emerge out of bracts per day was calculated using the following equation:


$$\begin{array}{l} Silking\;rate\;\left(silks\cdot d^{-1}\right)=\frac{90\%\;of\;the\;total\;silks\;\left(silks\right)}{The\;durations\;for\;90\%\;silk\;emergence\;\left(days\right)} \end{array}$$

### Grain Yield

Kernel row number, kernel number per row, and kernel weight were measured on matured ears from least three independent biological replicates. Kernel weight was determined on each ear after drying at 75 °C to constant weight. Ear yield was calculated with 14% water content.

### Statistical Analysis

All experiments involved at least three biological replicates from individual plants. Microsoft Excel 2010 and SPSS 26.0 were used for data collation, standardization, and statistical analysis. Diagrams were made by Origin 2018, Adobe Illustrator 2019, and R Studio. Significant differences were determined using the least significant difference test (*p* < 0.05).

## Results

### Hybridization and Classification of Hybrids With the Slender or Stubby Ears

**Figure 2:**
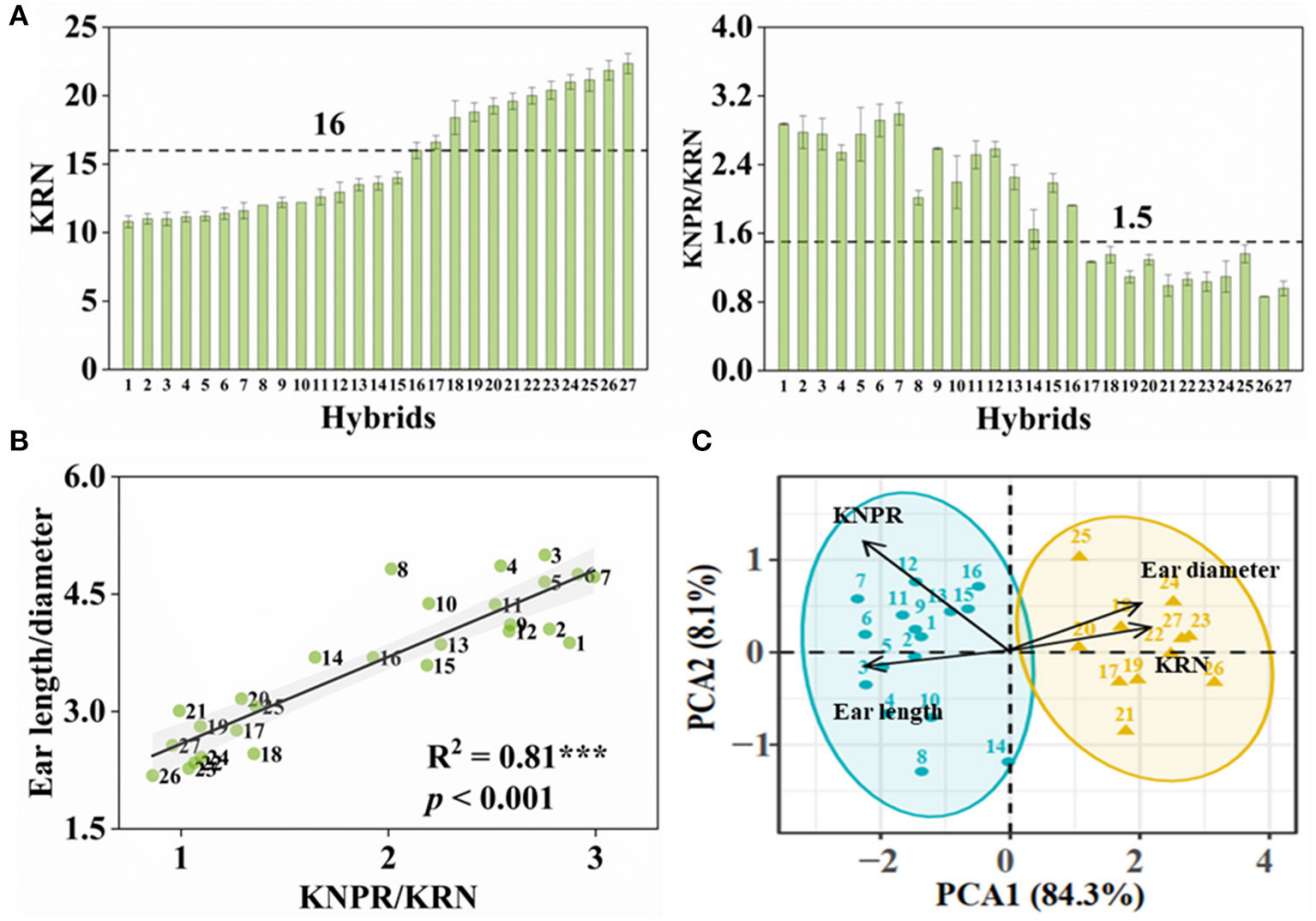
Classification of ear types in maize hybrids. **(A)** Kernel row number (KRN) and kernel number per row/kernel row number (KNPR/KRN) of maize hybrids. Ears with KRN < 16 and KNPR/KRN > 1.5 were defined as the slender ears. **(B)** Correlation analyses between KNPR/KRN and ear length/diameter. The gray background represents confidence intervals of 95%. **(C)** Principal component analysis (PCA) distinguished the hybrids with the slender or stubby ears into two distinct groups. ANOSIM statistic R = 0.9814; *p* = 0.001. Data correspond to 27 maize hybrids. Serial numbers of 1 to 27 were donated to these hybrids by order of KRN.

With purpose, we conducted crossing within inbred lines whose ears were with > or < 16 rows to obtain hybrids with more or less KRN, respectively. From these F1 hybrids, we obtained 27 hybrids with KRN ranging from 10.8 to 22.4 rows and KNPR ranging from 18.8 to 34.7 grains (Figure 2A and Supplementary Table S1). KNPR/KRN, significantly positively correlated with the ear length-diameter ratio (R^2^ = 0.81, *p* < 0.001) (Figure 2B), indicated the ear architectures of different hybrids. We set the thresholds of 16 rows and 1.5 KNPR/KRN, by which these hybrids were able to divide into two types, hybrids with the slender ears (whose KRN < 16 rows, KNPR/KRN > 1.5) or the stubby ears (whose KRN > 16 rows, KNPR/KRN < 1.5). We assigned serial numbers of one to 27 to these hybrids by order from least to most KRN, with hybrids 1–16 being classified as the slender ears and hybrids 17–27 as the stubby ears (Figure 2A). The principal component analysis (PCA) distinguished the hybrids with the slender and stubby ears into two distinct groups (ANOSIM statistic R = 0.9814, *p* = 0.001) (Figure 2C), validating the classifications of these hybrids with different ear architectures. The KRN and KNPR of parents of 27 hybrids are listed in Supplementary Table S1.

### The Stubby Ears Possessed More Silks and Faster Silking Rate Than the Slender Ears

The silking dynamics of the stubby and slender ears in response to water deficit have not been well understood. To this end, we applied water control from 1 to 7 days post-silking, exposing the whole period of silking to water deficit (Figure 1A). On the third day after first silk emerged, it was observed that SRWC, LRWC, and chlorophyll content of WD were significantly reduced compared with WW conditions (Figure 1B).

**Figure 3:**
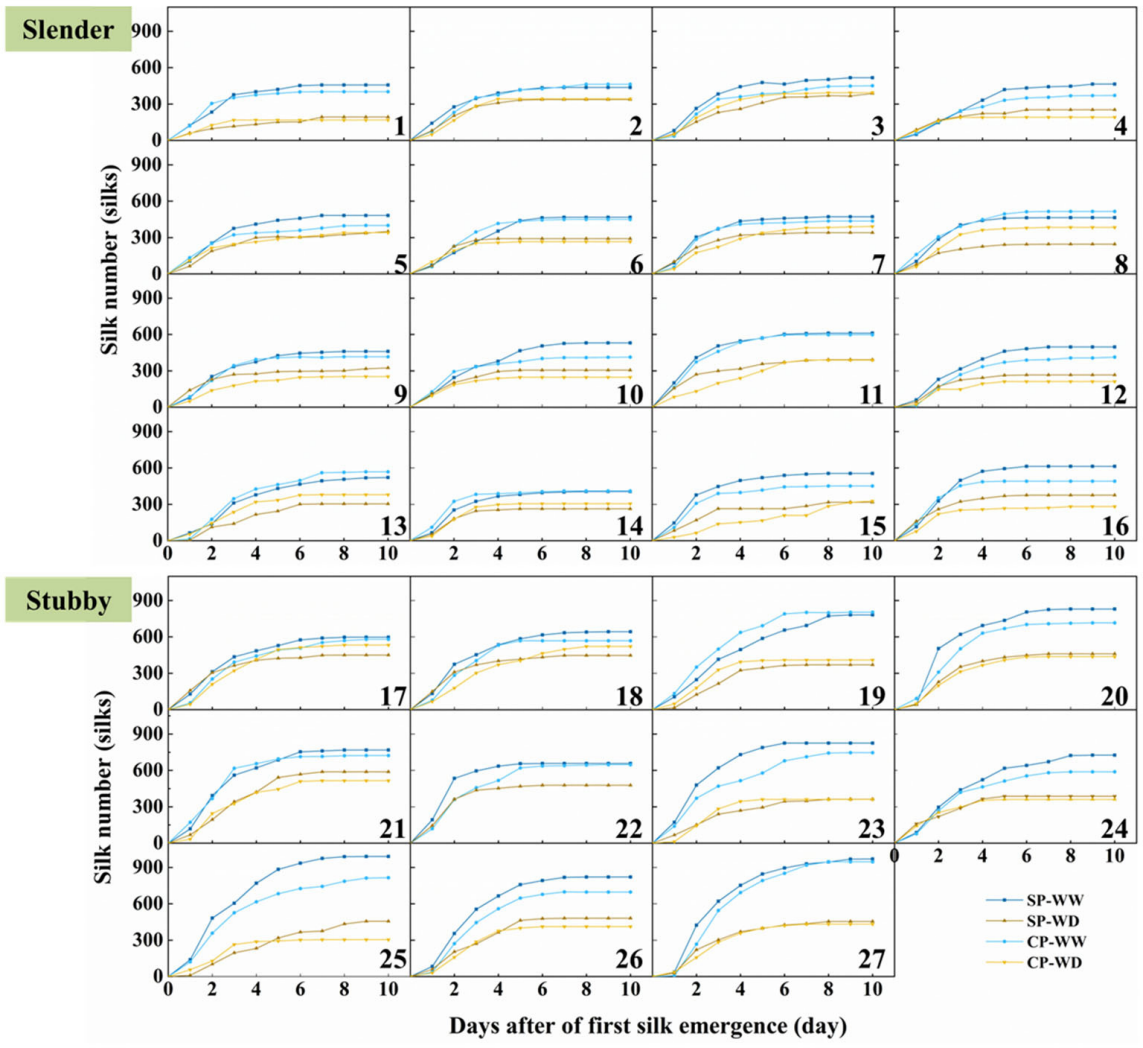
Silk emergence dynamics of 27 maize hybrids in different pollination and water treatments. Hybrids 1–16 are slender ear, and hybrids 17–27 are stubby ear. SP, synchronous pollination; CP, continuous pollination; WW, well-watered treatment; WD, water-deficit treatment.

Silking dynamics demonstrated that the silks extensively emerged in the first 3–4 days of silk emergence, and all plants stopped increasing before 7 days of silk emergence except hybrid combination 19 and 21 in SP-WW and 22 in SP-WD (Figure 3). WD treatment significantly reduced the silk number by 69 to 390 compared with WW (Figure 3). Interestingly, the eventual silk number of CP was more than the SP with or without water deficit (except hybrid combination 1, 14, 17, and 24 under WD treatment and 3, 8, and 21 under WW and WD treatments). Collectively, the eventual silk number in CP was 9.0 and 9.1% less than in the SP treatment in the slender and stubby ears under WW, respectively (Figure 3).

**Figure 4:**
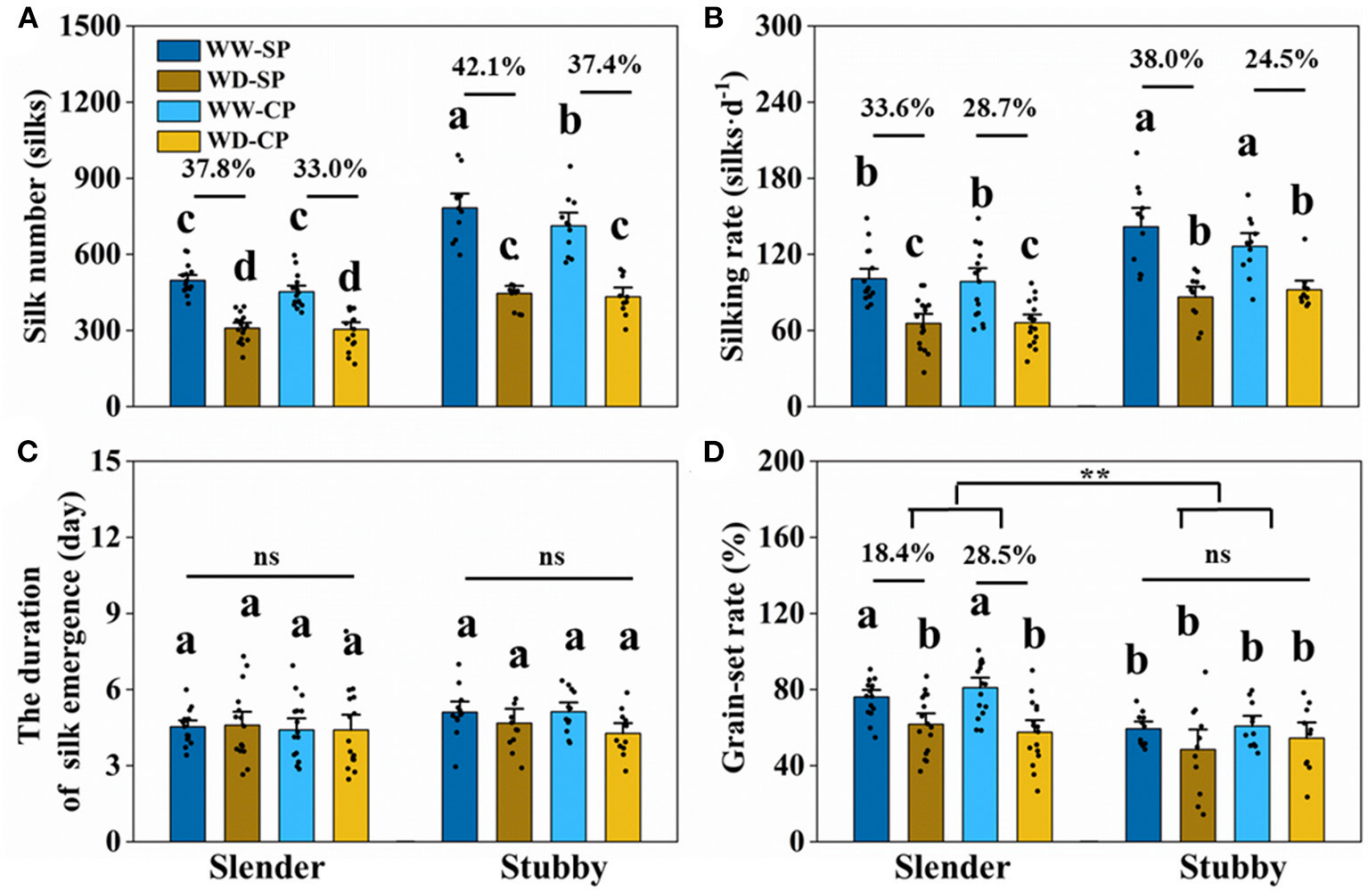
Silk number **(A)**, silking rate **(B)**, the duration of silk emergence **(C)**, and grain-set rate **(D)** of two ear types in different pollination and water treatments. One-way ANOVA followed by Duncan's new multiple range test, *n* ≥ 3; letters (a, b, and c) indicate significant differences (*p* < 0.05). Data represent the change rate of water-deficit treatments compared to well-watered treatments. Asterisks indicate that the loss rate between the slender and stubby ears was significantly different (*t*-test, *n* ≥ 3: **p < 0.01). d, day; ns, no significance; SP, synchronous pollination; CP, continuous pollination; WW, well-watered treatment; WD, water-deficit treatment.

The slender ears possessed less silk number than did the stubby ears (Figure 3), revealing an inherent difference of the two ear types. The silking dynamics and grain number demonstrated less silk number (371–614 vs. 568–991 silks), slower silking rate (61–149 vs. 84–200 silks·d^−1^), and a higher grain-set rate (55–91 vs. 49–74%) in the slender ears than the stubby ears (Figure 4). A similar difference was observed under WD treatment, for example, the slender ears possessed less silk number (168–395 vs. 304–589 silks) and slower silking rate (27–97 vs. 58–132 silks·d^−1^) compared with the stubby ears (Figure 4). However, there was no difference in durations for silk emergence in the two ear types with or without drought stress (Figure 4C). Similarly, there was no significant difference observed in the WD-induced losses in silk number, silking rate, or grain-set rate between the two ear types (Figure 4). Notably, the grain-set rates in the slender ears with SP and CP were reduced by 18.4% and 28.5% by WD, respectively, whereas they were not reduced by WD in the stubby ears (Figure 4D).

### Ear Yield

**Figure 5:**
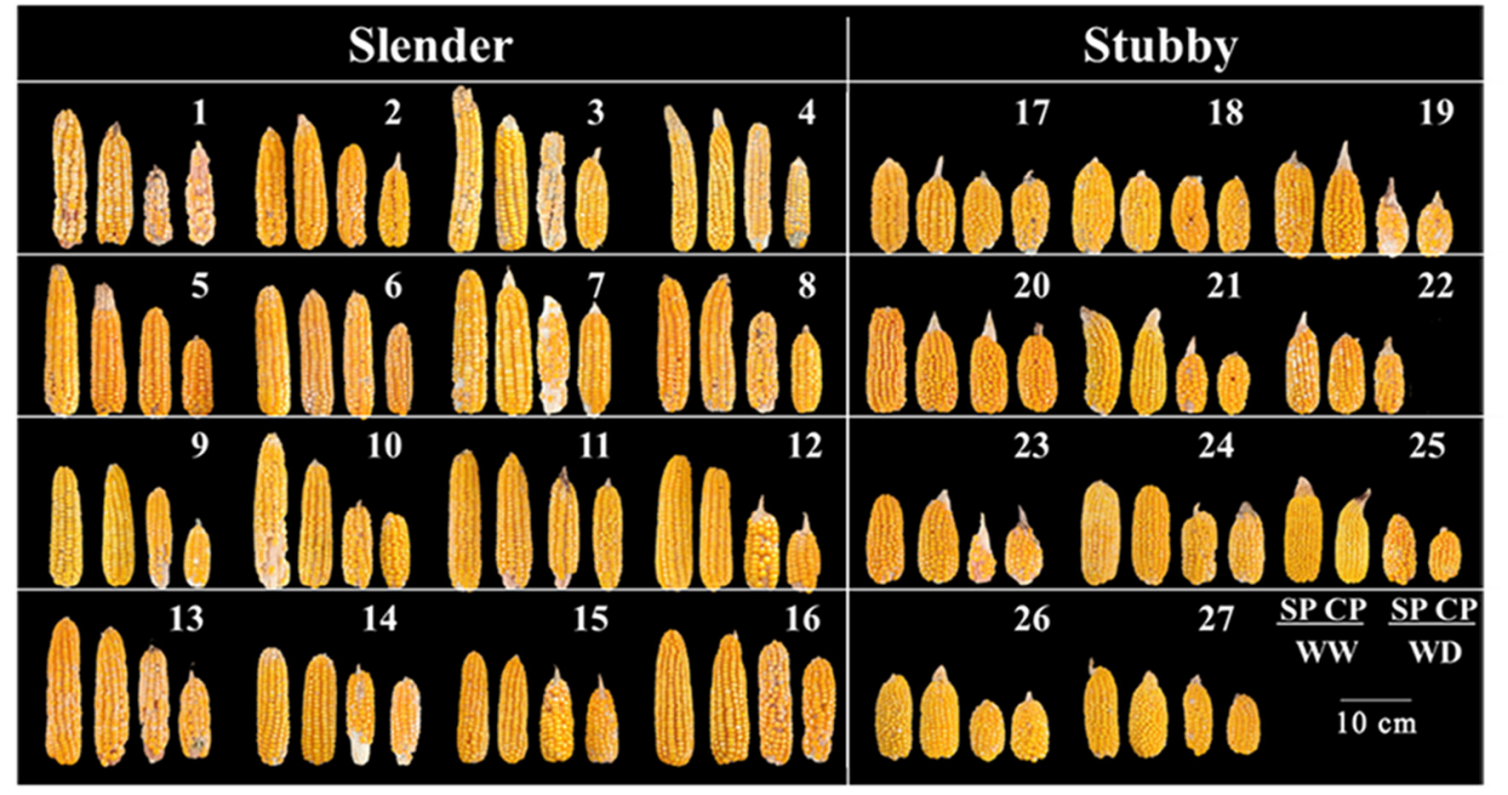
Ear phenotypes of 27 hybrids at maturity following pollination and water treatments at silking period. CP-WD of hybrid combination 22 was not included. SP, synchronous pollination; CP, continuous pollination; WW, well-watered treatment; WD, water-deficit treatment.

**Figure 6:**
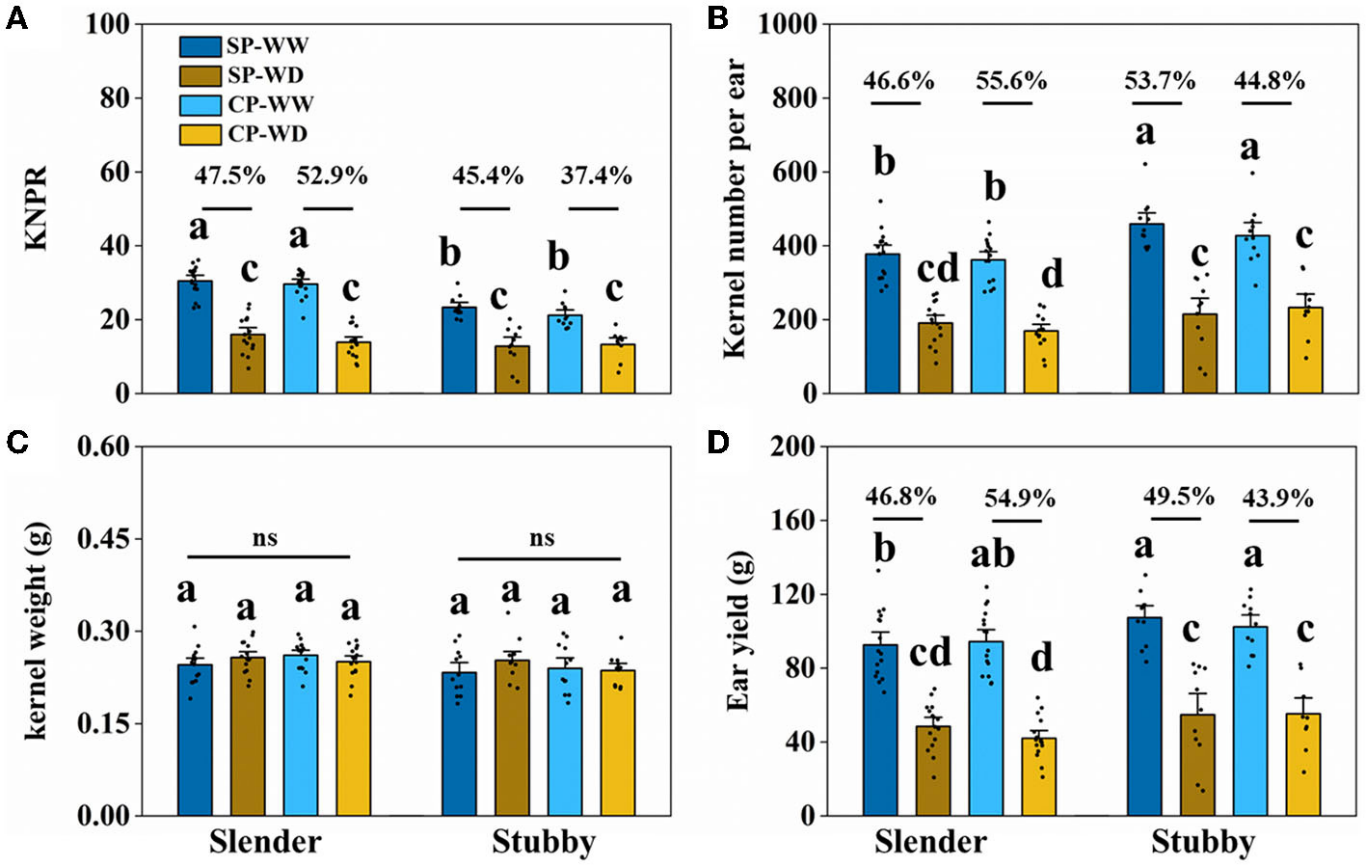
Kernel number per row (KNPR) **(A)**, kernel number per ear **(B)**, kernel weight **(C)**, and ear yield **(D)** of two ear types in different pollination and water treatments. One-way ANOVA followed by Duncan's new multiple range test, *n* ≥ 3; letters (a, b, and c) indicate significant differences (*p* < 0.05). Data represent the change rate of water-deficit treatments compared to well-watered treatments. ns, no significance; SP, synchronous pollination; CP, continuous pollination; WW, well-watered treatment; WD, water-deficit treatment.

The slender ears produced fewer kernels and yield compared with the stubby ears without regard to water condition (Figures 5, 6). Specifically, in WW, the slender ears possessed more KNPR (20.4–36.2 vs. 17.6–29.9 grains), less ear kernels (275–521 vs. 292–621 grains), and ear yield (66.9–132.8 vs. 80.9–130.4 g) than the stubby ears. Consistently, in WD, the slender ears possessed more KNPR (6.8–24.2 vs. 3.3–20.2 grains), less ear kernels (82–267 vs. 52–342 grains), and ear yield (20.7–68.8 vs. 13.7–82.2 g) than the stubby ears (Figure 6). Nevertheless, ear types or water conditions did not influence the grain weight (Figure 6C). These data revealed that WD decreased grain number and yield of both the slender and stubby ears, but there was no significant difference in the degree of reduction between the two ear types (Figure 6).

### When Silk and Grain Numbers Were Similar, the Stubby Ears Were More Resistant to Drought Stress Than the Slender Ears

These hybrids screened by ear types were inherently varied in silk numbers (ranged from 371 to 991), grain numbers (ranged from 275 to 621), and grain-set rate (ranged from 47 to 100%) in WW treatment (Figures 4, 6). These inherent differences may interfere with the assessment of drought resistance of ear architecture. To minimize the interfering effects, we expected to select those hybrids with different ear types but possessed similar sink capacities, for example, silk and kernel numbers, to further assess their responses to drought stress. We screened four slender (hybrids 11, 13, 15, and 16) and four stubby ears (hybrids 18, 20, 22, and 24), with both silk number (SP: 576 ± 22 vs. 655 ± 26; CP: 527 ± 34 vs. 596 ± 18) and kernel number (SP: 443 ± 26 vs. 448 ± 24; CP: 423 ± 17 vs. 405 ± 39) of the slender and stubby ears were not significantly different, for the subsequent analyses.

**Figure 7:**
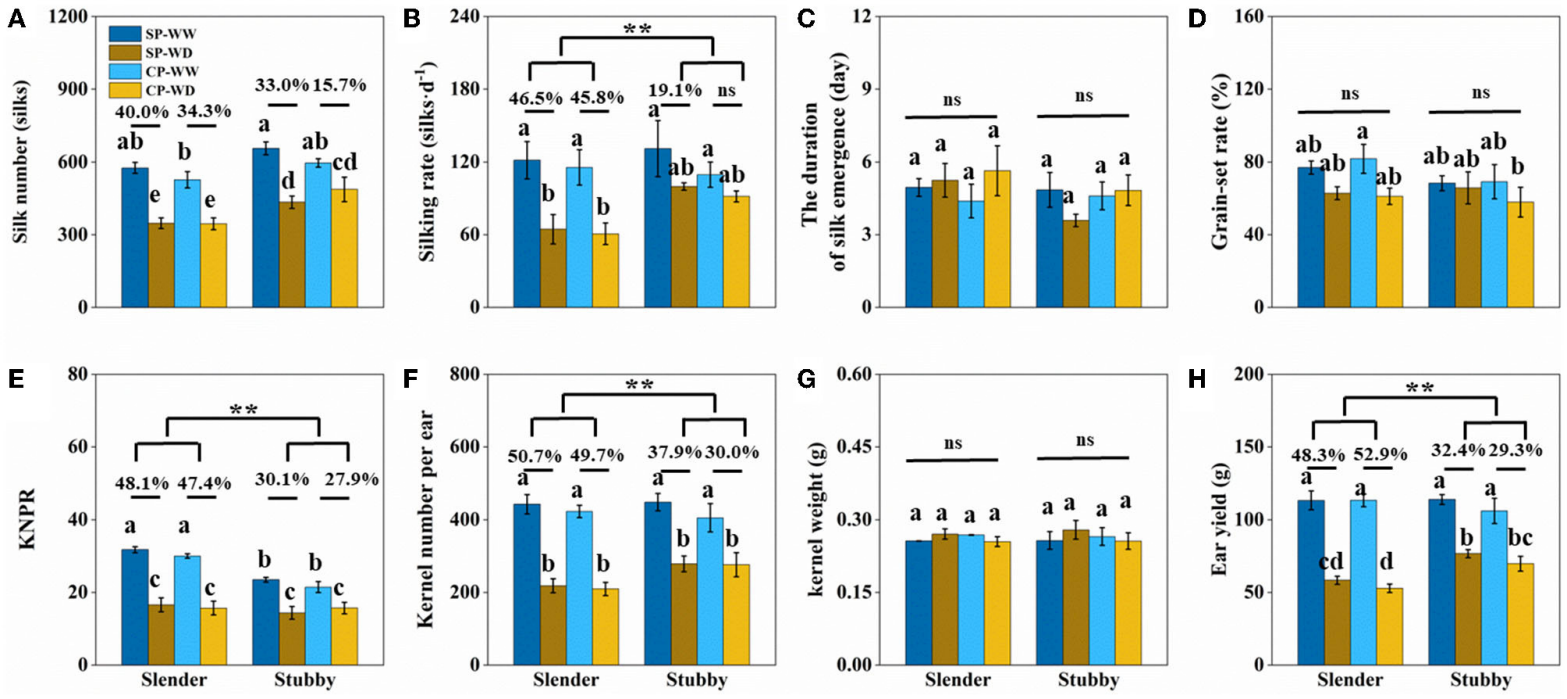
**(A–H)** Traits of silk and yield of two ear types (slender: hybrids 11, 13, 15, and 16; stubby: hybrids 18, 20, 22, and 24) with the similar silk number, kernel number per ear, and ear yield in the WW. One-way ANOVA followed by Duncan's new multiple range test, n ≥ 3; letters (a, b, and c) indicate significant differences (*p* < 0.05). Data represent the change rate of water-deficit treatments compared to well-watered treatments. Asterisks indicate that the loss rate between slender and stubby ear types was significantly different (*t*-test, n ≥ 4: **p < 0.01). d, day; ns, no significance; SP, synchronous pollination; CP, continuous pollination; WW, well-watered treatment; WD, water-deficit treatment.

WD significantly reduced silk number, but there was no significant difference in the degrees of reduction between the slender and stubby ears (Figure 7A). However, the silking rate losses in the slender ears were more severer than that in the stubby ears (SP: 46.5 vs. 19.1%; CP: 45.8 vs. 8.8%) (Figure 7B). In addition, the losses of KNPR (SP: 48.1 vs. 30.1%; CP: 47.4 vs. 27.9%) and losses of kernel number per ear (SP: 50.7 vs. 37.9%; CP: 49.7 vs. 30.0%) in the slender ears were significantly higher than that in the stubby ears (Figures 7E,F). Consequently, the slender ears possessed higher ear yield losses (SP: 48.3 vs. 32.4%; CP: 52.9 vs. 29.3%) than the stubby ears (Figure 7H). Notably, the duration of silk emergence, grain-set rate, and kernel weight had no significant difference in all treatments (Figures 7C,D,G).

### The Relationships Between Ear Architecture With Silking Rate and Yield

**Figure 8:**
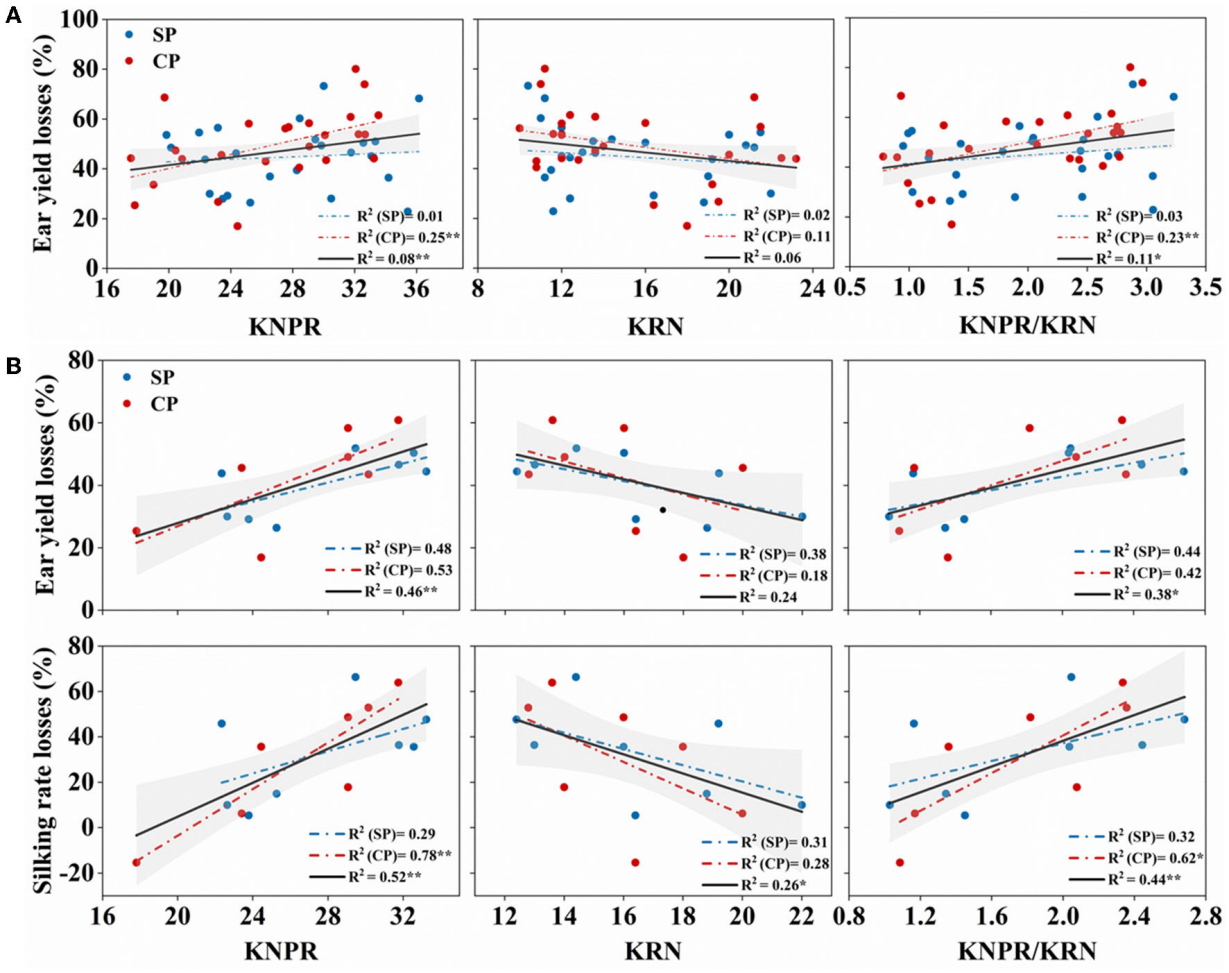
**(A)** Correlation between ear traits and ear yield losses in 27 hybrids; **(B)** correlation between ear traits and ear yield losses and silking rate losses in the eight hybrids with similar sink capacity. The red dot represents synchronous pollination (SP) treatments, and the blue dot represents continued pollination (CP) treatments. The gray strip represents confidence intervals of 95%. * and ** indicate significant differences at 0.05 and 0.01 levels, respectively. KNPR, kernel number per row; KRN, kernel row number.

Yield performance under drought stress reflects the resistance of varieties. Overall, in the 27 hybrids, the KNPR/KRN, positively correlated with the ear length-to-diameter ratio (Figure 2C), an indication of ear architecture, showed significantly positive correlations with the WD-induced yield losses (R^2^ = 0.11, *p* = 0.018) (Figure 8A). However, its correlations with the WD-reduced silk number and silking rate were not significant (data not shown). Interestingly, in the eight hybrids with similar sink capacities, KNPR (R^2^ = 0.46, *p* = 0.005) and KNPR/KRN (R^2^ = 0.38, *p* = 0.013) were significantly positively correlated with the WD-induced ear yield losses (Figure 8B). In addition, KNPR (R^2^ = 0.521, *p* = 0.002), KRN (R^2^ = 0.263, *p* = 0.050), and KNPR/KRN (R^2^ = 0.442, *p* = 0.006) were significantly correlated with the WD-reduced silking rate losses (Figure 8B). These results suggested that ear architecture were related with drought resistance, and the stubby ears had a faster silking rate to stabilize yield under drought stress.

## Discussion

Ear traits, kernel number per row (KNPR) and kernel row number (KRN), of maize have been concerned by researchers (Matsuoka et al., 2002; Chen et al., 2021, 2022; Ning et al., 2021), however, the influence of ear architecture on drought resistance is not well understood. In this study, we obtained 27 hybrids with the stubby or slender ears and applied manual pollination, including continuous and synchronous pollination, to observe the silking dynamics and evaluate drought resistance in two ear types. Our results showed that silk number, silking rate, and thereby kernel number were decreased under WD, resulting in yield loss. Unexpectedly, the slender and stubby ears demonstrated no significant difference in silk number losses and ear yield losses. To minimize the interference from inherent differences in silk number and sink capacities among different hybrids, those slender and stubby ears (four hybrids for each type) with the closest silk number, kernel number, and yield were selected for evaluation of drought resistance. We found that the stubby ears has the less silking rate losses, resulting in the less kernel number losses and ear yield losses in water deficit, in comparison with the slender ears. In addition, ear traits (KNPR, KRN, and KNPR/KRN) had significant correlation with silking rate losses and ear yield losses. Briefly, the stubby ears has the faster silking rate and the stronger resistance compared with the slender ears when the silk number and grain number are close. These findings emphasize the importance of ear architecture in silking and kernel set exposing to drought stress, providing new theoretical reference for breeders to select drought-resistant hybrids.

### Drought Stress-Reduced Silking Rate Contributes to Kernel and Yield Losses Irrespective to Ear Types

Silks is attached to the ovary receive pollen grains only when they emerged out of the bracts in maize. However, rapid growth of silks at flowering period, as indicated by accompanied silk elongation, weight accumulation, and high expressions of genes involved in cell expansion, is susceptible to drought (Fuad-Hassan et al., 2008; Oury et al., 2016a,b; Turc et al., 2016; Liu et al., 2021). Drought stress-inhibited silk growth at flowering may reduce kernel number and thus profoundly limits yield. Specifically, suppressed silk growth may extend anthesis-silking interval (ASI), leading to pollination failures (Grant et al., 1989; Edmeades et al., 2000; Fuad-Hassan et al., 2008; Benchikh-Lehocine et al., 2021). In addition, it may increase the PTGs within an ear, which is one of the main factors inducing post-fertilized kernel abortion (Shen et al., 2018). Besides, some of the silks could not emerge out of bracts to be pollinated under drought stress (Otegui et al., 1995; Oury et al., 2016b). In our study, silk number, silking rate, and kernel number were all reduced by WD irrespective to ear types (Figures 3, 5). Further analyses demonstrated that silk number and silking rate at flowering stage were significantly positively correlated with kernel number (R^2^ = 0.60, *p* < 0.001; R^2^ = 0.45, *p* < 0.001) and yield (R^2^ = 0.48, *p* < 0.001; R^2^ = 0.41, *p* < 0.001), respectively (Supplementary Figure S1), supporting that yield loss induced by flowering drought stress was attributed to silk growth. To support our findings, previous studies suggested that the sequential development and expansive growth, rather than sugar metabolism, in silks and ovaries were early influenced by drought and thus accounted for kernel abortion and yield loss (Oury et al., 2016b). Collectively with our findings on 27 hybrids with different ear architectures, we propose that faster silking rate, that is, less time gaps of silk emergence, could be a target for maize breeders and farmers to improve yield under drought.

### The Stubby Ears Had Stronger Drought-Resistant and Yield Potential Than the Slender Ones

**Figure 9:**
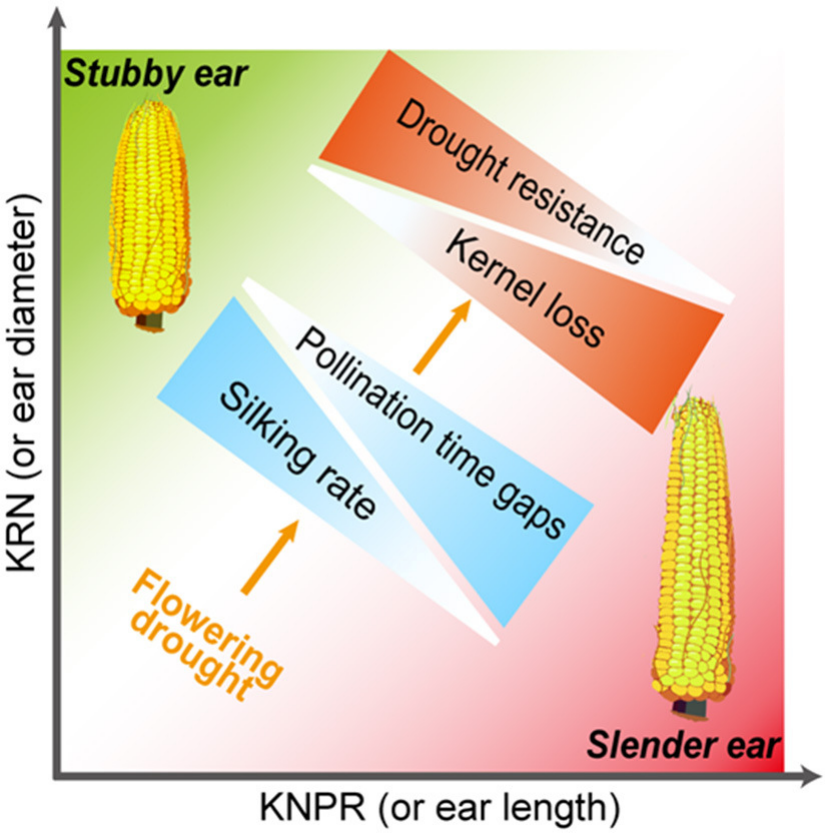
Hypothetic model demonstrating an ear architecture to ameliorate the yield losses induced by asynchronous silking rate and pollination time gaps. With similar numbers of silk and grain in well-watered, those ears with stubby architecture (more KRN but less KNPR) possess faster silking rate and likely less pollination time gaps than did those with slender architecture (less KRN but more KNPR), leading to less kernel losses of the stubby ears when exposing to flowering drought. These results suggest that the stubby ears are more resistant to drought compared with those slender ears. KRN, kernel row number; KNPR, kernel number per row.

Interestingly, when the inherent silk number and kernel number were similar, the stubby ears had more stable silking rate and yield than the slender ears under drought (Figure 7). Considering the sequential development of the ovary and silk of different cohorts along an ear follows a base-to-apex pattern (Oury et al., 2016b; Shen et al., 2018), the stubby ears with less ear length represents inherent advantage in silking rate. Indeed, less reduction in silking rate and kernel number under WD treatment were observed in the stubby ears (Figure 7), supporting the inherent advantage of the stubby ears under stress condition. Furthermore, the statement is supported by the significantly positive correlations between ear architecture (KNPR/KRN) and the WD-reduced silking rate (R^2^ = 0.44, *p* = 0.006) among the selected hybrids (Figure 8). Drought causes 15~20% of maize grain yield losses each year, and the situation is getting worse (FAOSTAT, 2010). A convenient strategy for farmers is selecting those varieties with stronger drought resistance. Although the physiological responses to drought stress are complex and often unpredictable, in maize, one of the major effects from drought stress is the inhibition of silk expansion (Borrás et al., 2007; Muhammad et al., 2015). Based on our result, we propose an ear architecture to ameliorate the yield losses induced by asynchronous silking and PTGs: the stubby ears has faster silking rate and stronger resistance compared with the slender ears when the silk number and grain number are close (Figure 9).

Noteworthy, the interest of traits for breeding would therefore depend on the correlation with yield (Araus and Cairns, 2014). Although most of the breeder is less aware of the relationships between ear architecture and drought resistance, they pay close attention looking at genes that control ear length and kernel row number, potentially valuble to improve ear architecture (Bommert et al., 2013; An et al., 2019; Du et al., 2021; Ning et al., 2021). Especially in recent years, great progress has been made in the study of controlling genes for ear traits (Du et al., 2021; Ning et al., 2021; Chen et al., 2022). In their studies, increases in ear length or kernel row number significantly promoted the yield per ear, but maize plants have limited photosynthetic capacity, especially under stress, which means that the kernel number cannot increase indefinitely. Therefore, the trait of stubby ear should be considered in the current varieties, which ultimately improve drought resistance of maize plants and contribute to food security.

## Data Availability Statement

The original contributions presented in the study are included in the article/Supplementary Material, further inquiries can be directed to the corresponding authors.

## Author Contributions

SS, S-LZ, and Y-BW conceived experiments. F-YL, SS, and S-LZ designed experiments. Y-BW provided parent materials, conducted hybridization, and collection of F1 seeds. F-YL and X-MC performed experiments and collected and analyzed the data. X-MC wrote the manuscript. SS and S-LZ helped with revisions. All authors contributed to the interpretation of the results, approved the final version of this manuscript, and agree to be held accountable for the content.

## Conflict of Interest

The authors declare that the research was conducted in the absence of any commercial or financial relationships that could be construed as a potential conflict of interest.

## Publisher's Note

All claims expressed in this article are solely those of the authors and do not necessarily represent those of their affiliated organizations, or those of the publisher, the editors and the reviewers. Any product that may be evaluated in this article, or claim that may be made by its manufacturer, is not guaranteed or endorsed by the publisher.

## Supplementary Material

Supplementary Figure S1 Correlation between yield traits and silk traits in all treatments. SP, synchronous pollination; CP, continuous pollination; WW, well-watered treatment; WD, water-deficit treatment. ^***^Indicates significant differences at 0.001 level.

Supplementary Table S1 Kernel row number (KRN) and kernel number per row (KNPR) of parents of maize hybrids used in this study.
