# Supplementary material for: Stubby or Slender? Ear Architecture Is Related to Drought Resistance in Maize
Source: Front Plant Sci. 2022 Jun 13;13:901186. doi: 10.3389/fpls.2022.901186 (PMC9235860; doi:10.3389/fpls.2022.901186)
Supplement: Supplementary Figure S1 — Correlation between yield traits and silk traits in all treatments. SP, synchronous pollination; CP, continuous pollination; WW, well-watered treatment; WD, water-deficit treatment. ***Indicates significant differences at 0.001 level. [file Data_Sheet_1.pdf]

Table S1. The kernel row number (KRN) and kernel number per row (KNPR) of parents of maize hybrids used in the present study.

Hybrids (F1)	KRN	KNPR	Female parent	KRN	KNPR	Male parent	KRN	KNPR
1	10.8	31.0	XL21	14	23	95C189-2	8	26
2	11.0	30.5	KS 2	12	22	95C189-2	8	26
3	11.0	30.3	KS 4	12	22	95C914-3	10	23
4	11.2	28.3	KS 4	12	22	95C545-2	12	16
5	11.2	30.8	KS 2	12	22	92C0003-10	10	22
6	11.4	33.2	XL21	14	23	92C0003-10	10	22
7	11.6	34.7	H2671	16	24	95C914-3	10	23
8	12.0	24.2	XL21	14	23	95C369	10	21
9	12.2	31.6	KS 2	12	22	95C914-3	10	23
10	12.2	26.8	XL21	14	23	95C914-3	10	23
11	12.6	31.7	XL21	14	23	95C545-2	12	16
12	13.0	33.4	KS 2	12	22	95C545-2	12	16
13	13.5	30.4	XL21	14	23	95C453-2	12	16
14	13.6	22.4	H2671	16	24	95C545-2	12	16
15	14.0	30.6	KS 2	12	22	95C453-2	12	16
16	16.0	30.8	H2671	16	24	95C453-2	12	16
17	16.6	20.8	L 598	22	23	94C1626-1	18	28
18	18.4	24.9	D 360	22	24	94C1368-1	18	34
19	18.8	20.5	D 598B	20	22	94C1349-3	20	32
20	19.3	24.9	D 598B	20	22	94C1626-1	18	28
21	19.6	19.4	94C1546-1	24	31	94C1374-2	22	26
22	20.0	21.3	94C1476-2	24	22	94C1374-2	22	26
23	20.4	21.1	D 598B	20	22	94C1368-1	18	34
24	21.0	23.0	D 360	22	24	94C1349-3	20	32
25	21.2	28.8	94C1546-1	24	31	94C1626-1	18	28
26	21.9	18.8	94C1546-1	24	31	94C1368-1	18	34
27	22.4	21.4	94C1546-1	24	31	94C1349-3	20	32

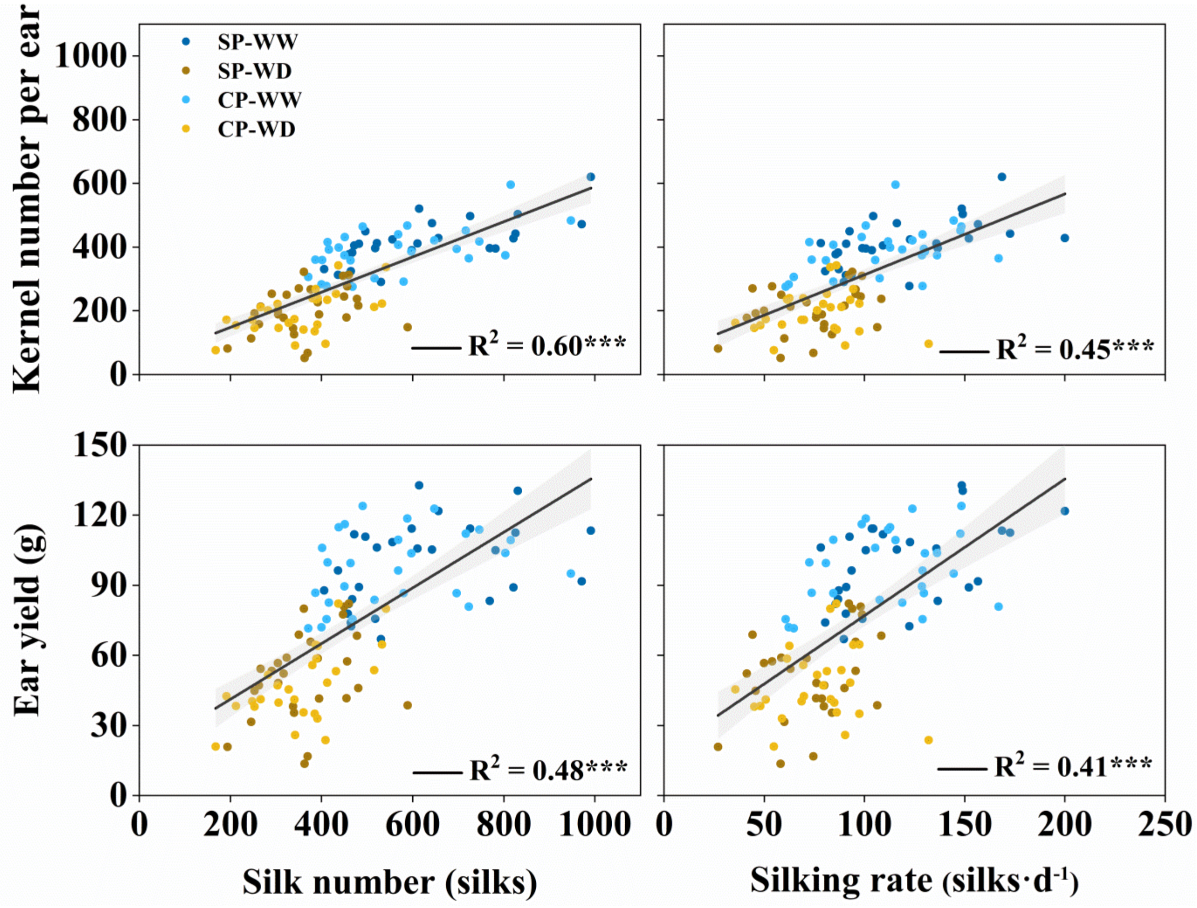

Fig. S1. Correlation between yield traits and silk traits in all treatments. SP: synchronous pollination, CP: continuous pollination, WW: well-watered treatment, WD: water-deficit treatment. ***indicated significant differences at 0.001 level, respectively.
